# Modeling the surface topography dependence of friction, adhesion, and contact compliance

**DOI:** 10.1557/s43577-022-00468-2

**Published:** 2023-01-18

**Authors:** Martin H. Müser, Lucia Nicola

**Affiliations:** 1 Department of Materials Science and Engineering, Saarland University, Saarbrücken, Germany; 2 Department of Industrial Engineering, University of Padua, Padua, Italy; 3 Department of Materials Engineering, Delft University of Technology, Delft, The Netherlands

## Abstract

The small-scale topography of surfaces critically affects the contact area of solids and thus the forces acting between them. Although this has long been known, only recent advances made it possible to reliably model interfacial forces and related quantities for surfaces with multiscale roughness. This article sketches both recent and traditional approaches to their mechanics, while addressing the relevance of nonlinearity and nonlocality arising in soft- and hard-matter contacts.

**Graphical abstract:**

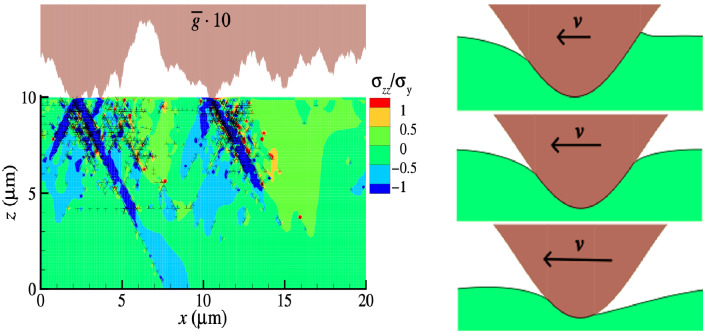

## Introduction

In his pioneering work on kinetic friction between solids, Coulomb^1^ argued that its physical cause must originate from either the interlocking (*l’engrenage*) of asperities—which can only be released through deformation, rupture, or by the raising of some summits over others—or by the coherence that interfacial molecules adopt due to their proximity and therefore needs to be overcome to produce motion. Coulomb’s assessment certainly contains the most information in the fewest words on the microscopic processes occurring in tribological contacts and largely summarizes how friction mechanisms are still categorized.^2^ However, it cannot be used to quantify interfacial forces, not even to predict trends such as whether roughness increases or decreases friction. More roughness generally leads to more plastic and viscoelastic deformation and thus to more energy loss, but it can also reduce the contact area and adhesive or capillary forces, which lowers friction. A qualitative picture cannot explain either why the three empirical solid friction laws are so frequently observed and what causes their breakdown when they fail. According to them, solid friction is approximately (1) proportional to load, but (2) independent of the apparent contact area and kinetic friction is less than static friction, but otherwise (3) independent of velocity.^3^ The simplicity of these laws, which can be augmented with the Archard–Reyes Law of wear stating that the volume of removed debris is proportional to the work done by friction,^4^ should not be taken as a sign that there are universal reasons for their validity or their breakdown. Nonetheless, it turns out that the surface topography of the bodies in contact and their change with load and sliding are indeed crucial. Hence, a proper characterization of surface topographies is essential;^5^ see also the recently posed surface topography challenge.^6^

**Figure 1:**
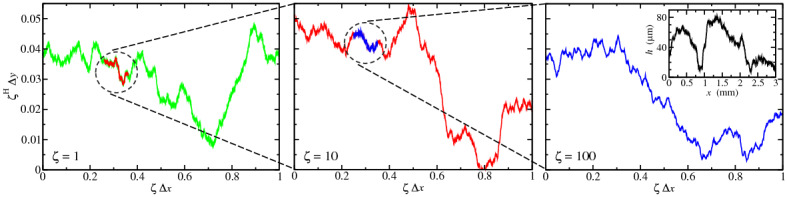
Computer generated, self-similar surface with Hurst exponent $H=0.8$ at different magnifications and a root-mean-square gradient of ${\bar{g}}=1$ at the finest scale. The inset shows experimental data of a $H \approx 0.75$ surface, which was produced for Reference 5. Great Britain’s west coast has the same fractal dimension as a one-dimensional, $H\approx 0.75$ surface.^11,12^

The (average) height spectrum of many freestanding surfaces,^7–9^ in particular those obtained after fracture or sandblasting,^10^ can be cast as

1 $$\begin{aligned} C({\mathbf {q}}) \propto \left\{ 1 + (q/q_\text {r})^2\right\} ^{-1-H}, \end{aligned}$$

where *C*(*q*) is the absolute square of the Fourier transform ${\tilde{h}}({\mathbf {q}})$ of the surface height, ${\mathbf {q}}$ is a wave vector, *q* its magnitude, and $q_\text {r}$ is the so-called roll-off wave vector. *H* is called the Hurst exponent. It generally lies between zero and one, usually $H \lesssim 1$. At very large *q*, *C*(*q*) must be cut off, ultimately because nature truncates roughness at the atomic scale. The approximate power-law dependence of the height spectra for $q_\text {r} \lesssim q < q_\text {atomic}$ makes the surface topography self-similar on small length scales. Surface profiles then look statistically similar to the eye when rescaling the height with a magnifying factor $\zeta ^{1/H}$ when in-plane coordinates are magnified with $\zeta$, as shown in **Figure** **1**. In real space, the squared height deviation from a given point ${\mathbf {r}}$ increases with increasing distance according to $\langle \left\{ h({\mathbf {r}}) - h({\mathbf {r}}+\Delta {\mathbf {r}})\right\} ^2 \rangle \propto \Delta r^{2H}$. A process closely related to randomly rough surfaces is the random walk leading to Fick’s diffusion, which can be described with a Hurst exponent of $H = 0.5$.

It is rather straightforward to exploit the mathematical description of self-similar surfaces in models describing their contact mechanics. This has been achieved with great success for linearly elastic, nonadhesive bodies. However, systematic extensions to topographies not obeying the random-phase approximation remain scarce. Likewise, attempts to account rigorously for nonlinearity and nonlocality as they arise due to plastic deformation of nominally flat surfaces or the coupling between viscoelasticity and adhesion are rather new. Some of the recent developments will be detailed in the following sections after a brief review of the state of the art of contact models and their limitations.

## Rough contact models and their limitations

Greenwood and Williamson (GW)^13^ pioneered the attempts to account for the effect that microscopic random roughness has on the mechanics of nominally flat surfaces. Their model assumes asperities to have a given radius of curvature and a Gaussian height distribution and, most critically, to act independently of each other. Once an asperity touches a (rigid) counterface, it deforms according to single-asperity characteristics (e.g., Hertzian contact mechanics in the original elastic GW model, but later modifications included adhesion^14^ and perfect plasticity^15^). The respective laws are then used to relate the deformation of the asperity, the force acting on it, and its true contact area with the counterface.

An intriguing prediction of many GW-inspired “bearing-area models” when applied to random, nonadhesive surfaces is that their real contact area often turns out to be (quasi) linear in the load *L* at small ratios of true and nominal contact areas, $a_\text {r}=A/A_0$, irrespective of the local asperity law (elastic or elastic-perfectly plastic) so that the mean pressure in true contact, $p_\text {c}=L/A$, is approximately constant. Despite their generic failures, which will be touched upon further below, rigorous simulations of rough, elastoplastic, and nonadhesive contacts using *J*2 plasticity,^16^ as well as dislocation dynamics simulations,^17^ confirm that *A* is linear in *L* at small $a_\text {r}$. Corrections are at most logarithmic in *L*, as long as the true contact contains a statistically significant number of microscopic contact patches.

Assuming the local interfacial shear stress $\tau _\text {s}$ to increase linearly with local pressure *p* squeezing two surfaces against each other,

2 $$\begin{aligned} \tau _\text {s} = \tau _0 + \upalpha p, \end{aligned}$$

leads to Amonton’s Law (i.e., to the linearity between the friction force $F = \tau _\text {s} A$ and normal load *L*). Here, $\tau _0$ and $\upalpha$ are system-dependent parameters, in addition to the proportionality factor linking *A* and *L*. Thus, under the given assumption, the friction coefficient $\upmu \equiv F/L$ assumes a constant value of

3 $$\begin{aligned} \upmu = \upalpha + \tau _0/p_\text {c} \end{aligned}$$

at small $a_\text {r}$, where $p_\text {c}$ is the load-insensitive ratio of load and true contact area.

To what extent Equation 2 is reasonably accurate or highly flawed certainly depends on the system of interest. The behavior of simple boundary lubricants (i.e., very thin layers of lubricants), keeping hard surfaces from intimate mechanical contact turns out to be consistent with Equation 2, as can be seen from simulations of generic bead-spring polymers confined between atomically smooth surfaces^18^ or from experiments of single-asperity contacts, in which the adhesion between two curved mica surfaces is screened through the use of appropriate electrolytes.^19^

In these cases, $\tau _0$ can be loosely associated with an adhesive (offset) stress and $\upalpha$ can be given a geometric interpretation in terms of hard-sphere interactions. However, Bowden and Tabor’s^3^ original use of Equation 2 in their attempt to rationalize friction coefficients for metal-on-metal contacts may be too simplistic.^20^ They related $\tau _\text {s}$ and $p_\text {c}$ to the shear strength and flow strength (or hardness) of the two metals in contact, respectively. This poorly reflects the scale dependence of plasticity and real hardening laws, even if the hardness of materials and its dependence on grain size allows important guidelines for the friction coefficient in contact between metals to be rationalized, as Chandross and Argibay neatly summarized recently.^20^ Other nonlocal dissipation mechanisms such as viscoelastic losses cannot be reconciled with Equation 2 either, as will be discussed in the section/paragraph on nonlocal effects. Nonetheless, Amonton’s Law may still hold when Equation 2 is violated. One explanation would be that the increase of contact area with load at small nominal pressures is mainly due to a rescaling of the prefactor of the contact-patch-size distribution rather than to the extension of its tail to larger patch areas. Thus, predicting contact-patch distributions correctly is a critical requirement for a quantitative contact mechanics approach.

**Figure 2:**
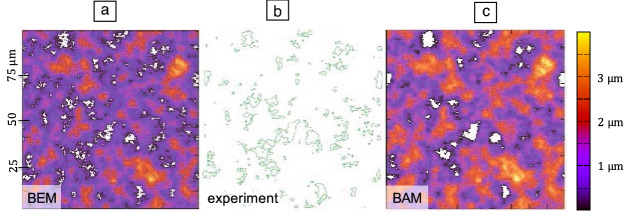
Gap and contact topographies obtained at relative contact area at $\approx 3\%$ in the contact-mechanics challenge.^21^ Panel (a) shows the gap obtained using a boundary-element method (BEM), (b) contact lines obtained experimentally with a total internal reflection method using a printed, scaled-up surface, and (c) the gap deduced from a generic bearing-area model (BAM). White color indicates contact in (a) and (c), whereas black circles highlight a detail of the contact area to facilitate comparison. Reproduced with permission from Reference 21 except for panel (c), which was drawn for this article.

One reason why bearing-area models (BAMs) are problematic is that they neglect the elastic coupling between asperities (i.e., they ignore that surface points near the highest peak are pushed down much more than distant points). As a consequence, BAMs overestimate the mean gap between solids and predict contact to be too localized near high peaks, as revealed in **Figure** **2**. It compares the contact topography formed by an elastic solid and a rigid, randomly rough, computer-generated surface as obtained in (a) a rigorous boundary-element method (BEM), (b) an experiment bringing an elastomer in contact with a 3D printed version of the surface, and (c) a generic BAM assuming the highest $3\%$ of the indenter points to be in contact. The generic BAM misses many of the small patches revealed both experimentally and in rigorous simulations, in particular to the right of the black circle. Moreover, BAM leads to distincly less rugged contact edges than numerically rigorous approaches.

A new approach to contact mechanics^22^ (see Persson’s summary of his theory in this issue^23^) also finds contact area to be linear in load at small $a_\text {r}$, but it remains accurate beyond the linear regime. Specifically, for frictionless elastic surfaces, it predicts contact area to obey

4 $$\begin{aligned} a_\text {r} = \mathrm {erf}\{\sqrt{\pi }p/(E^* {\bar{g}})\}, \end{aligned}$$

where $E^* = E/(1-\nu ^2)$ is the contact modulus of the solid (when both bodies are deformable, their inverse contact moduli add, as in a series coupling of springs), *E* is the Young’s modulus, $\nu$ the Poisson’s ratio of the elastic material, and ${\bar{g}}$ is the (combined) root-mean-square (rms) height gradient of the surfaces. Although the theory was originally derived for ideal random roughness (see Equation 5), it makes astonishingly accurate predictions on the relative contact area if ${\bar{g}}$ is averaged only over the true contact area,^24^ even in the limiting case of single-asperity contacts, which are the polar opposite to ideal random roughness.

**Figure 3:**
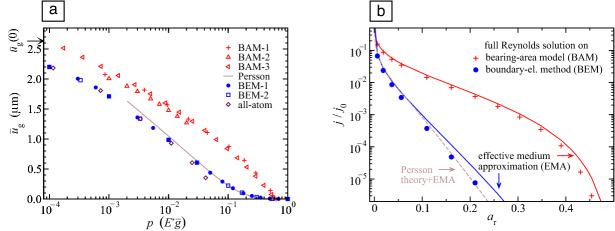
(a) Mean gap as a function of pressure for three bearing-area models (BAMs, red symbols), Persson’s contact mechanics theory (gray line), two rigorous boundary-element methods (BEMs, blue symbols), and a scaled-down all-atom model (open diamonds). Adapted from Reference 21. (b) Relative leakage current $j/j_0$ versus relative contact area $a_\text {r}$ for topographies computed with a BAM and a BEM. Symbols represent full solutions of the Reynolds equations, whereas lines assume an effective medium approximation (EMA) to it. Adapted from Reference 29.

Although Persson’s contact mechanics theory is not exact, it generally predicts central interfacial properties much more accurately than BAMs. For instance, the probability of having contact a distance $q_\text {s}^{-1} \lesssim \Delta r \lesssim q_\text {r}^{-1}$ away from a point of contact, decays with $1/\Delta r^{1+2H}$ in Persson’s theory.^25^ This agrees with numerical results of a rigorous BEM, whereas a generic bearing-area model finds a faster decay according $1/\Delta r^{2+2H}$.^26^ Another example for the accuracy of Persson’s theory is its ability to predict how the mean gap, ${\bar{u}}_\mathrm {g}$, decreases with increasing load.^27^ This was also revealed in the contact mechanics challenge, where Persson’s prediction agreed with the results of numerically rigorous BEMs, whereas GW-inspired models agreed with each other, but not with the correct reference, as demonstrated in **Figure** **3**a. Rescuing BAMs is possible,^28^ but requires the elastic coupling between contact spots as well as their coalescence to be included, which is arguably a more complex task than to simply code or run a BEM.

Failing to predict the mean gap as a function of load is particularly detrimental for the estimation of leakage, as described by the Reynolds thin-film equation, which assumes the local resistance to fluid flow to increase with the inverse third power of the gap. Traditional BAMs easily overestimate leakage by several orders of magnitude even far away from the percolation threshold, as can be seen in Figure 3b.^29^ In contrast, the Reynolds flow can be estimated quite accurately using an effective medium theory taking the gap distribution function from either BEM, Persson’s theory, or even experimental data, acquired, for example, via digital image correlation,^30^ as input.

An important property to deduce from the dependence of the mean gap on pressure is the contact compliance defined as $\chi = -\partial {\bar{u}}/\partial p$, or its inverse the better-known contact stiffness. $\chi$ turns out to be proportional to first estimates of the interfacial resistance to heat flow and electric current, as well as to the interfacial shear compliance.^31,32^ This is because for elastic solids the mentioned properties can be calculated in similar ways from similar second-order partial differential equations so that proportionality coefficients are merely products or ratios of materials constants.

The complete system can be seen as a series coupling of solid A, the interface, and solid B, whose respective compliances or resistances add up to a combined value. However, corrections are needed to obtain accurate estimates for the conductivities. Radiative heat transfer, mainly through evanescent waves, add to the heat conductance, while oxide layers or other layers adsorbed on top of metals, increase the electric resistance.^32^ Estimating the pertinent corrections requires the gap distribution or the contact area to be known, which can be deduced from quantitative theories and simulations or even from experiments.

Thus, although bearing-area models provide an intuitive framework with which trends can be rationalized, Persson’s theory is quantitative. However, Persson’s theory has been rigorously tested predominantly on elastic solids and indenters with “ideal” random roughness. Although we expect it to be applicable (potentially with appropriate modifications) to other systems, the need for quantitative tools remains. At present, computer simulations are our best chance to model rough interfaces with a small number of uncontrolled approximations.

## Computational approaches to roughness

To model surface topography effects numerically, height profiles must be acquired first. Ideally, though unlikely, a friendly experimentalist willingly shares artifact-free data defined on a large matrix. In the real word, modelers fall back to computer-generated virtual surfaces, which can have the advantage to be periodic thereby allowing finite-size or (hyper-) surface effects to be minimized. There are many different ways to generate height functions, $h({\mathbf {r}})$, representative of randomly rough surfaces, the simplest one being to set their (complex) Fourier coefficients to

5 $$\begin{aligned} {\tilde{h}}({\mathbf {q}}) \propto \sqrt{C({\mathbf {q}})} \exp \{2\pi \mathrm {i} w({\mathbf {q}})\}, \end{aligned}$$

where $w({\mathbf {q}})$ is an independent, uniform random number on [0, 1].

Surfaces generated with Equation 5 produce, on average, a Gaussian height distribution. However, machined or worn surfaces, as those described in the accompanying article by Aghababaei et al.^33^ tend to have skewed height distributions, because tops get flattened while valleys are less affected by plasticity. To reproduce simultaneously height distributions and spectra, a surface can be set up producing the correct spectrum, then the *n*th highest point be assigned the height that the *n*th highest point (of a discretized surface) is supposed to have, where *n* runs through all point indices. The resulting surface is Fourier transformed, its spectrum rescaled to the target spectrum, and the procedure iterated until deviations from the target are tolerable.^34^ Other constraints violating the random-phase approximation can certainly be realized in a similar fashion.

In the simplest interaction model, surfaces are assumed to be impenetrable, or alternatively, one can use a quickly increasing overlap potential emulating finite-range repulsion and, if applicable, adhesive interactions. Next, the surface displacements must be related to the stresses acting on the surface. A point force acting normally onto a surface of a semi-infinite, linearly elastic solid leads to a displacement that decays as 1/*r* from the point of action. This dependence can be expressed quantitatively through the equation

6 $$\begin{aligned} {\tilde{\sigma }}({\mathbf {q}}) = -q {E^*} {\tilde{u}}({\mathbf {q}})/2 \end{aligned}$$

in terms of the Fourier transforms or coefficients of stress, ${\tilde{\sigma }}({\mathbf {q}})$, and surface displacement field, ${\tilde{u}}({\mathbf {q}})$. Equation 6 can be generalized in many different ways so that in addition to normal displacement and stresses, other effects^35^ can be considered such as those due to finite thickness^36^ and viscoelasticity.^37^ Polonsky and Keer^38^ pioneered the fast Fourier transform (FFT)-based solution of contact problems by exploiting the sparseness of the stress–displacement coupling represented in Equation 6. In their original approach, the stresses at the nodal points in real space were continuously adjusted to yield a value of zero in noncontact while satisfying the nonoverlap constraint. Later investigations^35–37^ assume the displacements to be dynamic degrees of freedom that either relax to the minimum in the fastest possible way, or propagate according to viscoelastic properties, which can be achieved by coupling each ${\tilde{u}}({\mathbf {q}})$ mode to an appropriate set of Zener or related rheological elements.^37^ Although BEMs are typically limited to linear (visco-) elasticity, they can be coupled to discrete dislocation dynamics (DDD) allowing plasticity, as described in DDD, to be included effectively.^39^ Moreover, a recent reformulation of the Mindlin fundamental solution in a Fourier representation allowed continuum plasticity to be described at a similar complexity as with Fourier-accelerated BEMs, that is, with a numerical complexity scaling as ${\mathcal {O}}(N\ln N)$, where *N* is the number of surface grid points.^40^

The type of questions that can be addressed within the previously described methods include (1) do two rough surfaces deform elastically so that they—using Coulomb’s words—form a coherence that needs to be overcome to initiate sliding, (2) what contact stiffness $\kappa$ does a mechanical interface produce, or (3) at what point does roughness “kill” adhesion? Succinct answers in the framework of linearly elastic bodies and ideal random roughness would be (1) yes, but if elastic asperity interlocking were the only friction mechanism, friction coefficients would be tiny;^41,42^ (2) $\kappa$ would be approximately linear in the nominal pressure *p* at small *p* and increase roughly exponentially with *p* at large *p*^27,43^—these trends can be deduced (in parts visually) from the data shown in Figure 3a—and (3) when the reduced surface energy ${\tilde{\gamma }}=\gamma /v_\text {ela}^\text {fl}$ falls below approximately 0.5,^44^ where $\gamma$ is the surface energy and $v_\text {ela}^\text {fl}$ is the elastic energy in full contact per unit area.^44^

Answer (3) may require additional explanation. The transition between high and low adhesion is rather abrupt only for short-range adhesion and precise values remain difficult to predict.^44,45^ However, it can be noted that true contact at zero or marginally tensile loads occurs dispersed across a nominally flat interface if ${\tilde{\gamma }} \gtrsim 0.5$, but typically only in the vicinity of the highest peak when ${\tilde{\gamma }} \lesssim 0.5$.^44^ We leave it up to the reader, so to speak as a bonus problem, to demonstrate that the latter finding is consistent with the observations that large asteroids, which are made up of (large!) fractured rubble particles having a density close to that of silicate rocks (i.e., $\approx 2$ t/$\hbox {m}^3$) have a natural upper spinning period exceeding 2.3 h. An elaborate master solution to the problem is given in Reference 46.

Similar questions as those just answered for ideal elasticity still wait for an answer when the contacting bodies are more complex, for example, when their energy dissipation occurs internally and not only in the immediate vicinity of the contact or in a boundary lubricant.

## A critical challenge: Nonlocal effects

As already mentioned, a point force acting on the surface of a semi-infinite, linearly elastic body, be it normal or parallel to the surface, leads to a displacement, which merely decays with the inverse distance from that point. This long-range deformation is at the root of many complications, including the inappropriateness to formulate contact mechanics as a theory of variables that can be defined as averages of local quantities such as rms height ${\bar{h}}$ or rms height gradient ${\bar{g}}$. Whereas the contact area of repulsive, linearly elastic contacts can be estimated reasonably well from Equation 4 having the (resolution-dependent) ${\bar{g}}$ as the only topographic parameter, the elastic energy is a (weighted) sum or integral over wave vectors. Such sums are needed when calculating, for example, $v_\text {ela}^\text {fl}=(E^*/4)\sum _{\mathbf {q}} q \vert {\tilde{h}}({\mathbf {q}}) \vert ^2$ (valid for a frictionless contact) or the load–displacement relation in Persson’s theory.^47^ They cannot be reduced to averages over locally defined variables such as ${\bar{h}}^2$ or ${\bar{g}}^2$, for which the prefactor $E^*/4$ in the $v_\text {ela}$ sum would have to be omitted and the term *q* in the summand be replaced with 1 and $q^2$, respectively.

The just-mentioned nonlocality is at the root of why dissipative or irreversible processes are frequently also nonlocal and moreover scale-dependent, in which case a frequently stressed argument for the validity of empirical friction laws, specifically Equation 2, can be problematic. For example, in an initially elastic description of contact between two metals, maximum shear stresses, or rather maximum deviatoric stresses, $\max (J_2)$, occur a certain distance away from the interface, so that plastic deformation—assuming $J_2$ plasticity to be valid—is not triggered by the deviatoric stresses $J_2$ at the interface but in the bulk.^48^ A potential reason for meaningful deviations from classical friction and wear laws can arise when the superposition of subsurface stress fields of adjacent contact spots becomes significant (i.e., large enough to trigger the deep propagation of subsurface cracks), as observed in atomistic simulations during the transition from mild to severe wear.^49^

**Figure 4:**
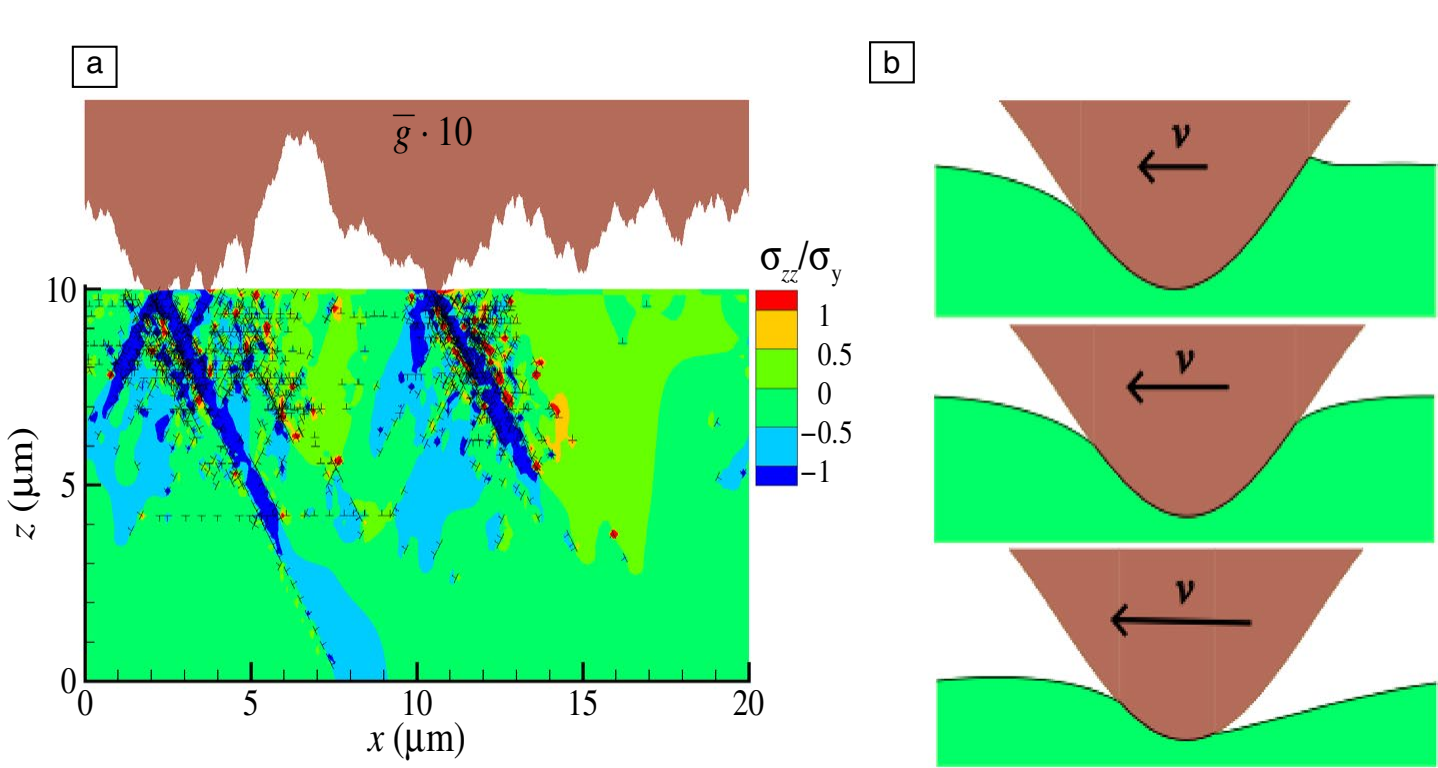
Examples of processes leading to dissipation outside the interface. (a) A metal crystal indented by a rough, rigid surface: plastic shear bands, broader than the contact areas, extend deep into the material. Black T-symbols represent dislocations. The rough indenter is stretched in the $z$-direction by a factor 10 to better visualize its roughness. Adapted from Reference 17. (b) A sinusoidal adhesive indenter sliding past a viscoelastic foundation. The sliding velocity increases from top to bottom, while the viscoelastic response of the foundation transitions from short- to long-ranged. Courtesy of N. Menga and G. Carbone.

When a detailed plasticity description is needed at scales too small for continuum plasticity to be applicable, it must be kept in mind that plastic deformation in crystalline metals is carried by dislocation glide, which has two important implications. First, plasticity requires not only $J_2$ to exceed a critical value, but also the presence of defects serving as dislocation sources. It is the random distribution of these defects in the body that can lead to a nonsymmetric plastic response, even when the loading is symmetric. Second, the Peach–Koehler force drives dislocations away from the region, where they were generated.^17^ As a consequence, plastic deformation is not confined to the region where high stresses occur and can result in complex shear bands, as those depicted in **Figure** **4**a. Their generation requires energy, whereby they contribute to dissipation during indentation, but also during sliding. Interaction between extending shear bands leads to additional superposition of subsurface stress fields, and increases the risk of crack nucleation at the intersection between bands, where dislocations form strong junctions and stress release through plasticity is hindered.

When contacts are soft and adhesive, nonlocal effects arise due to the interplay between adhesion and viscoelasticity. In adhesive soft-matter rolling^50^ or sliding,^51^ dissipation is due to the difference between the energy gained when the contact closes at its leading edge, and the energy lost when the contact opens at its trailing edge. Depending on the loading speed, significant dissipation can take place far away from the contact edges,^52^ which can be viewed as crack tips. This is because the viscoelastic solid acts like a soft elastic solid both in the immediate vicinity of the crack and far away from it, while the viscous response dominates at intermediate distances from the crack tip,^52–54^ which led de Gennes to coin the term viscoelastic trumpet.^55^ The transition from short- to long-ranged viscoelasticity can be observed on the contact profile of the viscoelastic foundation in Figure 4b, where a sinusoidal tip slides at increasing velocities from top to bottom.

An important consequence of the dissipation due to a sliding adhesive contact is that the relaxation time of the system can be dramatically enhanced compared to the elastomer’s intrinsic relaxation times, in particular when the adhesion is short-ranged. This effect causes large demands on simulations, because reproducing the bulk viscoelasticity and crack dissipation simultaneously would require small interaction ranges and thus extremely fine discretization.

An additional nonlocal dissipation mechanism of rough surfaces arises from multistability (i.e., at a given mean relative distance between two solids, different microscopic surface configurations exist). A generic example would be a parabolic indenter with small-scale sinusoidal roughness.^56^ Individual, microscale asperities can discontinuously snap into or out of contact and they do not immediately jump back to their old position after tip velocity inversion. As for any other instability, the energy loss is approximately the difference between the potential energy just before the instability and a short time after it, that is, after the instability-induced vibrations have calmed down.^57^ This mechanism can lead to significant dissipation during quasistatic motion and, in the words of Coulomb, occurs whenever an interface has discontinuously adjusted the coherence of relevant degrees of freedom, whether they are molecular or coarse-grained (e.g., asperity-sized in nature).

**Figure 5:**
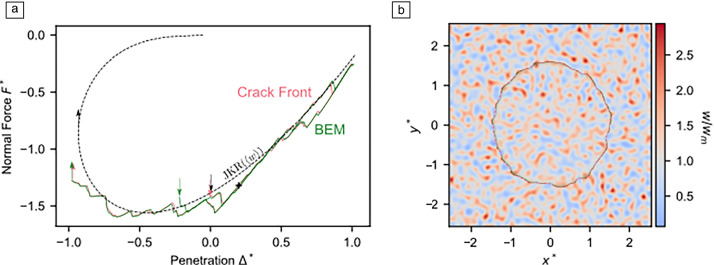
(a) Comparison of the force–displacement relation as obtained in a crack-front model, the Johnson-Kendall-Roberts (JKR) solution, and a full boundary-element method (BEM). (b) Visualization of the contact contour and the effective surface energy. From Reference 60.

The difficulty in modeling adhesive multistability lies in the need for short-ranged adhesion,^58^ which entails the necessity for an extremely fine discretization to avoid spurious effects.^59^ Sanner and Pastewka found a rather compelling solution to this problem for spherical indenters with small-scale roughness^60^ by mapping it onto a crack-front model with quenched disorder,^61^ which can prove useful to describe the motion of a contact line of a liquid droplet on a substrate.^62^ To this end, they first exploited the possibility to express the elastic energy of a singly connected contact domain as a function of its contour.^63^ Second, they mapped the effect of surface roughness onto a local surface energy by using an effectively fractional and thus nonlocal height gradient. Specifically, they interpreted the inverse Fourier transform of $\sqrt{qE^*/4}\,{\tilde{h}}({\mathbf {q}})$ as the square-root of a local energy density. Using this crack-front approach, BEM-based solutions could be reproduced quite closely. More importantly, they found a rather flat force–displacement curve on retraction, as is characteristic for spherical indenters with small-scale roughness, which are retracted from a soft, adhesive foundation,^64,65^ as seen in **Figure** **5**.

## Conclusions

Modeling phenomena involving surface topography-induced processes have come a long way, in particular, describing those that Coulomb envisioned to be responsible for the friction between solid bodies. What can be seen as particularly impressive is that there is an increasing number of accurate theoretical and computational tools that use as input materials properties, besides the surface topography, and give as output realistic predictions. This is certainly a breath of fresh air compared to the art of “post-diction,” where the modeling requires a significant fraction of the final result to be used as input. At present, not only the contact response of elastic bodies can be predicted with accuracy, but important steps forward have been made also in the modeling of both soft- and hard-matter contacts. The modeling of viscoelastic and of microscale metal contacts has brought forward the importance of nonlocal effects, namely dissipation processes occuring at a distance from the interface, and thus the relevance of explicitly modeling the solids.

Unfortunately, we could only scratch the surface and had to omit quite a few success stories, such as the experimental study reproducing the displacement field of the contact mechanics challenge to within roughly 10% of the rms-gap,^66^ detailed comparisons between theory or experiments of the elastoplasticity in spherical tips with microscale roughness,^67^ or the leakage through seals, which was at least touched upon in an article in this issue of *MRS Bulletin*.^23^

Fortunately, we’re not done yet. Despite all progress, we are not aware of studies successfully reproducing adhesive hysteresis when viscoelasticity and roughness-induced multistability both contribute substantially. It may sound simple, but we believe it to be a quite ambitious endeavor to not only match final displacement fields of real surfaces, but also their time dependence. Likewise, we are not aware of macroscale contact plasticity simulations where microscale effects are incorporated, and where the frictional response is an emergent behavior. Finally, as detailed in the article by Aghababaei et al.,^33^ the modeling of surface evolution has just started. Modeling of and comparing to or even better predicting experiments monitoring simultaneously topography changes and friction forces, as Korres et al.^68^ achieved, represents the ultimate challenge.

## Data Availability

No new data were generated for this article.
